# Multiomic analysis of non-glaucomatous human trabecular meshwork cells

**DOI:** 10.64898/2026.07.28.740161

**Published:** 2026-07-29

**Authors:** Anh H. Pham, Isabella G. Moceri, Zachary Batz, Nivedita Singh, Avinash Soundararajan, Katelyn Kane, Tutut Nurjanah, Emine Bilir, Rong Du, Yiqin Du, Milton A. English, Jennifer A. Faralli, Samuel Herberg, Stephanie How, Ruminder Preet Kaur, Susanna Li, Suhani Patel, Padmanabhan P. Pattabiraman, Carl M. Sheridan, Anand Swaroop, Ying Ying Sun, Neeru A. Vallabh, Sanjoy K. Bhattacharya, Donna M. Peters, Kate E. Keller

## Abstract

Glaucoma is an irreversible blinding disease that affects millions of individuals worldwide. Elevated intraocular pressure (IOP), regulated by the trabecular meshwork (TM) in the anterior eye, is the only modifiable risk factor. Human TM cells can be cultured from donor eyes, providing a precious resource for studying factors that induce or prevent glaucoma. The goal of this study was to produce datasets that define the molecular profile of human non-glaucomatous TM cells. Using 18 human TM cell strains cultured from non-glaucomatous individuals deposited from seven laboratories in the USA and UK, this study used transcriptomic, proteomic, lipidomic, and metabolomic analyses to characterize the molecular content of TM cells. The data herein provides the most comprehensive multiomic analyses of human TM cells to date and will be a useful resource for researchers and clinicians in the TM and glaucoma fields.

## Full Text Availability

The license terms selected by the author(s) for this preprint version do not permit archiving in PMC. The full text is available from the preprint server.

